# Supplementary material for: A semi-automated cell tracking protocol for quantitative analyses of neutrophil swarming to sterile and S. aureus contaminated bone implants in a mouse femur model
Source: PLoS One. 2024 Jun 20;19(6):e0296140. doi: 10.1371/journal.pone.0296140 (PMC11189170; doi:10.1371/journal.pone.0296140)
Supplement: S1 File — (DOCX) [file pone.0296140.s008.docx]

**A semi-automated cell tracking protocol for quantitative analyses of neutrophil swarming**

Sashank Lekkala^1,2^, Youliang Ren^2,3^, Jason Weeks^2^, Kevin Lee^1,2^, Allie Jia Hui Tay^1,2^, Bei Liu^2^, Thomas Xue^1,2^, Joshua Rainbolt^1,2^, Chao Xie^2,3^,

Edward M. Schwarz^1,2,3^ and *Shu-Chi A. Yeh^1,2,3^

^1^Department of Biomedical Engineering, University of Rochester, Rochester, NY, USA, ^2^Center for Musculoskeletal Research, University of Rochester Medical Center, Rochester, NY, USA, ^3^Department of Orthopaedics and Rehabilitation, University of Rochester Medical Center, Rochester, NY, USA

*Corresponding authors

[ShuChi_Yeh@URMC.Rochester.edu](mailto:ShuChi_Yeh@URMC.Rochester.edu)

[Edward_Schwarz@URMC.rochester.edu](mailto:Edward_Schwarz@URMC.rochester.edu)

**Supplementary Methods**

**Step-by-Step Protocols for Cell tracking using Trainable Weka Segmentation and TrackMate**

1. **Pre-processing**
2. Open the .tif file using Fiji.
   1. File > Open

Note: To perform all the pre-processing in one step, copy the macro (.ijm file, S1 Appendix) and the classifier (.model file, S2 Appendix) into the working directory. Run the plugin: Plugins > Macros > Run. Save the .tif file and skip to step 10.

1. Split the neutrophil (tdTomato^+^) and bacteria (ECFP^+^) channels.
   1. Image > Color > Split Channels
2. Duplicate the channel for processing.
   1. Image > Duplicate (Check: Duplicate stack) > OK
3. Convert the 3D stack to 2D using maximum intensity projection.
   1. Image > Stacks > Z Project. Select all the slices and Projection type: Max Intensity.
4. Correct drift using the Image Stabilizer plugin.(1)
   1. Plugins > Image Stabilizer. Use the default values and check ‘output to a new stack’.

Tip: If the Image Stabilizer plugin fails, the StackReg plugin(2) can serve as an alternative.

1. Generate a minimum intensity projection of this stabilized image. This step is critical to render background noise and stationary artifacts that are commonly present in vivo.
   1. Image > Stacks > Z Project. Select all the slices and Projection type: Min Intensity.
2. Subtract the minimum intensity projection from all the slices to remove stationary artifacts (Fig 1C). Save this stack.
   1. Process > Image Calculator. Image1 should be the stabilized stack and Image2 should be the minimum intensity projection image. Click ‘Yes’ for the ‘Process all 60 slices?’ pop-up.
3. Image segmentation using Trainable Weka Segmentation.(3)
   1. Plugins > Segmentation > Trainable Weka Segmentation.
   2. Using the ‘Load classifier’ option to open the classifier file. Then, click on ‘Get probability’ and wait for the probability maps to appear.
4. A hyper stack of probabilities with the first channel for background and the second channel for neutrophils will appear (the order of these channels depends on the order of labels in TWS) (Fig 1D).
   1. Extract the neutrophil channel. Image > Hyperstacks > Make Subset. Extract all the slices from Channels: 2.
5. **Cell tracking**(4,5)
6. Plugins > Tracking > Trackmate. In the TrackMate window, make sure the XY and T stack scaling is correct (pixel width, height, and time interval). If any details are incorrect, go to Image > Properties to correct them.
7. Next > LoG (Laplacian of Gaussian) detector > Next > Estimated object diameter: 16 micron, Quality threshold: 0.001, Enable ‘Pre-process with median filter’ and ‘Sub-pixel localization’ > Next.
8. Do not change anything in the ‘Initial thresholding’ and the ‘Set filters on spots’ windows. All the spots detected in step 11 should be included for tracking, as the image output from TWS has represented all the foreground objects.
9. Next > Select a tracker: LAP tracker >Next > Frame to frame linking: Max distance: 20 micron > Next.
   1. The maximum linking distance was set to 20µm based on previous manual tracking data and it depends on the speed of cells and the time interval of longitudinal imaging.
10. Set filters on tracks. Click on the green + icon to add parameters. Track displacement: Above, 16 (the size of the neutrophil); Track duration: Above, 60 (tracked in more than 3 frames) > Next (Fig 1E).
    1. To set the filter to a particular value, click on the left edge of the highlighted window and type the number.
11. Display options. These options can be changed according to user comfort for tracking. But when saving data, consistent display features should be used for easy visual comparison.
12. The progress can be saved by clicking on the ‘Save’ button which will be saved as a .xml file.
13. Overlay the tracks onto the preprocessed stack.
    1. Open the .xml file using a text editor software such as Notepad and find the line ‘ImageData’ using ctrl+F (Windows) or command+F (Mac) in the document.
    2. Replace the file name and directory. <ImageData filename="*Name of the file saved in step 7 with the file extension*" folder="*Directory of this stack*" width="483" …
    3. To load this file, go to Plugins > Tracking > Load a TrackMate file. This will open both the .tif and the TrackMate window.
14. **Tracks Verification**

Verifying the tracks is the only step in the protocol where the user interferes. Overall, this protocol outputs tracks with high accuracy (Fig 1E, 1F). However, in neutrophil-dense regions, the user may need to adjust the tracks more intensely through steps 18-21. Here we describe a few criteria (steps 22-24) to ensure accuracy and reproducibility.

1. To inspect and edit occasional tracking errors, click on the ‘TrackScheme’ button to open a window with individual track details. The color of the spots and the links will match the criteria set by the user in the ‘Display options’ window. Each track is assigned an ID which can be seen by hovering the mouse on the top frozen row. The left frozen column lists the corresponding frame number of the spot.

Note: The frame number in the TrackScheme window starts at Frame 0, so the frame number in the stack is one number ahead of the frame number in the TrackScheme window.

1. Clicking on a spot in the TrackScheme window will highlight it in the stack and vice versa. Right-click on a spot in TrackScheme to “Select whole track” or to “Remove spots and links” (for incorrectly tracked cells). To delete a link (for splitting a track), click on the connection between the spots and right-click on the selection and click “Remove spots and links”.
2. To connect tracks, click on the ‘Toggle linking’ icon. When it turns blue, click and drag to connect two spots.
3. To create a new spot, position the mouse at the desired location on the image and press the ‘A’ key. To change the position of this spot, position the mouse on the spot and hold the space bar and then move the mouse to a new location. Press the ‘E’ key to increase and ‘Q’ to reduce the radius of the spot.
4. Deleting an entire track or a portion of the track.
   1. Incorrect tracks (or portions of tracks) are easy to detect. The usual examples are tracks bouncing back and forth between two stationary cells.
5. Connecting two tracks: Follow the criteria in the order below to determine if two tracks need to be connected.
   1. Frame gap: If the second track begins near the end of the first track both spatially (<20µm radius) and temporally (single frame gap), then the two tracks can be connected.
   2. Intensity penalty: If there are a few potential candidates from the first criterion, to decide which tracks to connect, the user can penalize changes in intensity. That is, if the neutrophils in the two tracks have similar “brightness”, they can be connected.
   3. Direction penalty: Abrupt changes in the directionality can also be penalized. This penalty needs to be carefully implemented since in some cases, the neutrophils may not have a preferred direction of motion.
   4. Shape/size penalty: Changes in the shape or size of the neutrophil can also be penalized.
   5. In the cases where neutrophils cluster and cannot be individually segmented, end the existing tracks and start new tracks when the cells emerge from the cluster. Tracks can be manually connected as needed.
6. Using the criteria above, a portion of the track may need to be disconnected or deleted and connected to an appropriate track. If still unclear, leave the tracks unconnected. These penalties can also be mathematically implemented in TrackMate in step 13.
7. Edit all the tracks and make sure they are correct (see the Tracks Verification steps above) (Fig 1F). Once all the tracks are edited, close the TrackScheme window and save your progress.
8. Next > Next > Select an action. Capture Overlay > Execute > OK. This action creates a stack with overlaid tracks which can be saved as a .avi file. Export tracks to XML file > Execute (save in a specified folder) > OK.
9. **Post-processing and quantification**
10. Open the exported track file (usually saved as *Filename_tracks.xml*) using Microsoft Excel.
11. Make sure that column I is “/particle/#id” and column J is “/particle/@nSpots” since the MATLAB code is hard-coded to match this template (S3 Appendix). The two columns may be reversed in Windows and Mac OS systems.
12. Save this File as a .xlsx file.
13. Copy the MATLAB file into the working directory and open the code in MATLAB.
14. Click “Run”. If you have not changed the working directory in MATLAB, click on “change folder” in the popup.
15. A command will appear in the Command Window: “Input the file name”. Type the file name from step 26 with the extension and press enter.
16. The code will run and add two workbooks (Sheet 2 and Sheet 3) to the .xlsx file.
17. Sheet 2 contains the details of individual tracks at individual frames. Sheet 3 contains the summary details for each track such as total displacement, velocity, total distance, and speed (Fig 1G).

Note: The units of speed and velocity are in µm/frame. That is, if the frame gap is 30 seconds, the units of speed and velocity are in µm/30 sec.

1. The directionality can be calculated as displacement/distance to determine whether the cell motility is directional or in a random walk.

**Volume analysis using Imaris**

1. Open Imaris and click on the ‘Observe Folder’ option. Choose the directory containing the IV-MLSM video files.
2. Convert the files to native Imaris format (.ims) by double-clicking on each file.
3. Create a new Surface and select the neutrophil channel. Enable the ‘Smooth’ option, setting ‘Surfaces Detail’ to 1 µm.
4. Apply ‘Background Subtraction’ with a diameter of 4 µm.
5. Manually select an appropriate intensity threshold such that all the neutrophils are selected (S2B Fig). Use the same threshold across all the time points for a given animal.
6. Set the ‘Number of Voxels Img = 1’ to convert the number of voxels to volume. Then, use the ‘Number of Voxels’ histogram to set a size exclusion range to remove any artifacts (S2C,2D Fig).
7. Extract the neutrophil volume data from the statistics panel.
8. Normalize the neutrophil volume to the total volume of interest to obtain the percentage of volume occupied by neutrophils.

**References:**

1. Kang Li. http://www.cs.cmu.edu/~kangli/code/Image_Stabilizer.html. 2008. The image stabilizer plugin for ImageJ.

2. Thévenaz P, Ruttimann UE, Unser M. A pyramid approach to subpixel registration based on intensity. IEEE Transactions on Image Processing. 1998;7(1):27–41.

3. Arganda-Carreras I, Kaynig V, Rueden C, Eliceiri KW, Schindelin J, Cardona A, et al. Trainable Weka Segmentation: a machine learning tool for microscopy pixel classification. Bioinformatics [Internet]. 2017 Aug 1 [cited 2023 Aug 9];33(15):2424–6. Available from: https://dx.doi.org/10.1093/bioinformatics/btx180

4. Tinevez JY, Perry N, Schindelin J, Hoopes GM, Reynolds GD, Laplantine E, et al. TrackMate: An open and extensible platform for single-particle tracking. Methods. 2017 Feb 15;115:80–90.

5. Ershov D, Phan MS, Pylvänäinen JW, Rigaud SU, Le Blanc L, Charles-Orszag A, et al. TrackMate 7: integrating state-of-the-art segmentation algorithms into tracking pipelines. Nature Methods 2022 19:7 [Internet]. 2022 Jun 2 [cited 2023 Aug 9];19(7):829–32. Available from: https://www.nature.com/articles/s41592-022-01507-1

**Supplementary Figures**


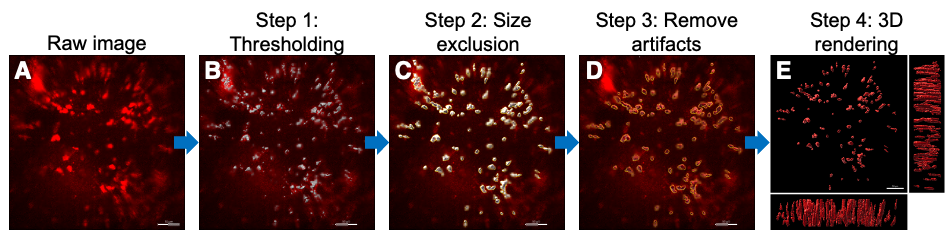


**S1 Fig. Workflow for volumetric quantification of neutrophil volume using Imaris.** (A) Representative 2D LIMB image from the raw Z-stack (20x, Scale bar = 50µm). (B) A user-defined threshold was applied to include all the neutrophils (shown in gray) (C) Features smaller than the average size of neutrophils (10-12µm) were excluded from the analysis. (D) Imaging artifacts were manually removed from the analysis (for example, the large fluorescent spot in the top left corner). (E) 3D rendering of the selected volume in X, Y, and Z planes.


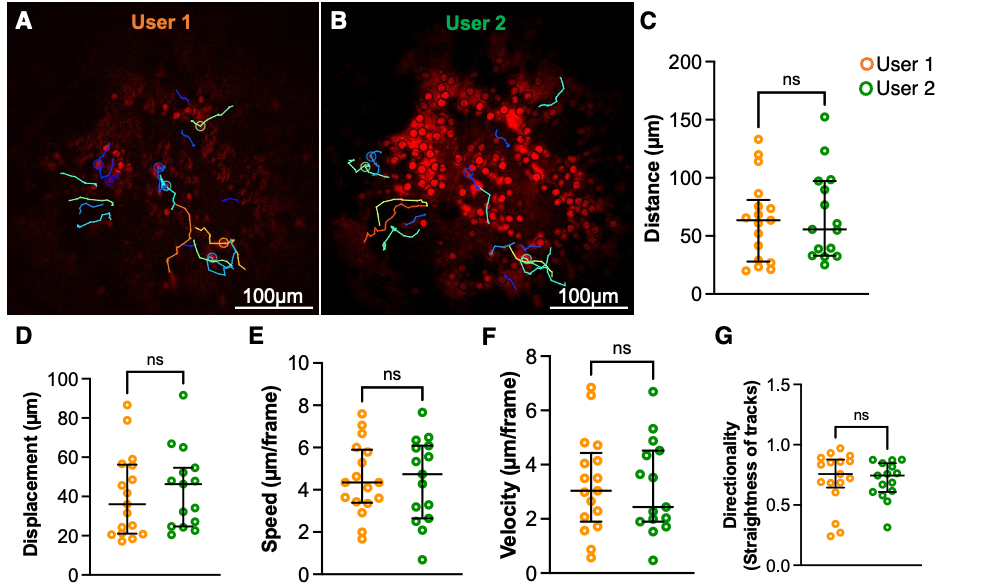


**S2 Fig. Inter-person variability before using the semi-automated protocol.** User 1 performed minimum intensity projection subtraction but used an arbitrary threshold to segment neutrophils resulting in poor cell detection. User 2 did not perform any pre-processing resulting in several non-moving artifacts impeding the ability to reliably track all moving cells. Representative 2D IV-MLSM image with overlaid tracks generated by user 1 (A) and user 2 (B). Note that users 1 and 2 identified different tracks due to differences in pre-processing and segmentation. Semiautomated quantification of neutrophil distance traveled (C), displacement (D), mean speed (E), mean velocity (F), and directionality (G), was performed and the data are presented with the median and interquartile range. (ns = not significant as determined by Mann-Whitney tests (n=15-17 tracks)).


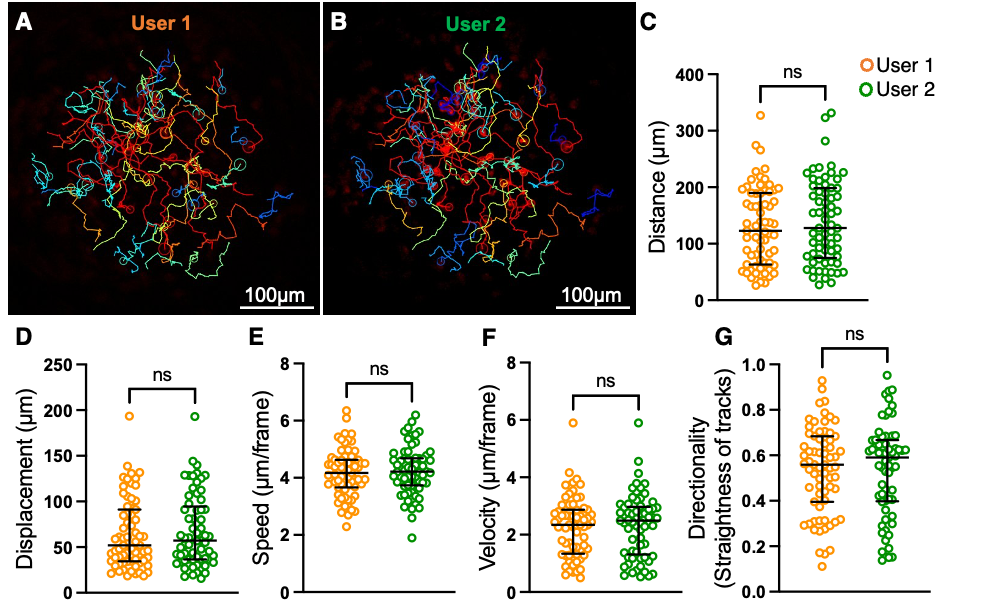


**S3 Fig. The tracking protocol resulted in low inter-person variability in generating tracks in the infected cases.** Two users independently analyzed IV-MLSM timelapse videos from an infected mouse and the generated track parameters were compared. Representative 2D IV-MLSM image with overlaid tracks generated by user 1 (A) and user 2 (B). Semiautomated quantification of neutrophil distance traveled (C), displacement (D), mean speed (E), mean velocity (F), and directionality (G), was performed and the data are presented with the median and interquartile range. (ns = not significant as determined by Mann-Whitney tests (n=67 tracks for user 1 and 65 tracks for user 2)).


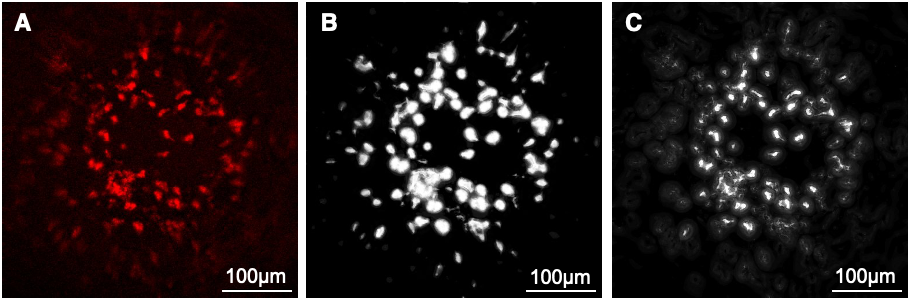


**S4 Fig. Comparison of two different TWS classifiers.** (A) Representative input image for TWS. The probability maps resulting from a conservative classifier (B) depicted larger neutrophils compared to the original image but detected all the neutrophils. On the other hand, an aggressive classifier (C) depicted retained the size of the neutrophils but failed to recognize faintly fluorescent neutrophils.

**
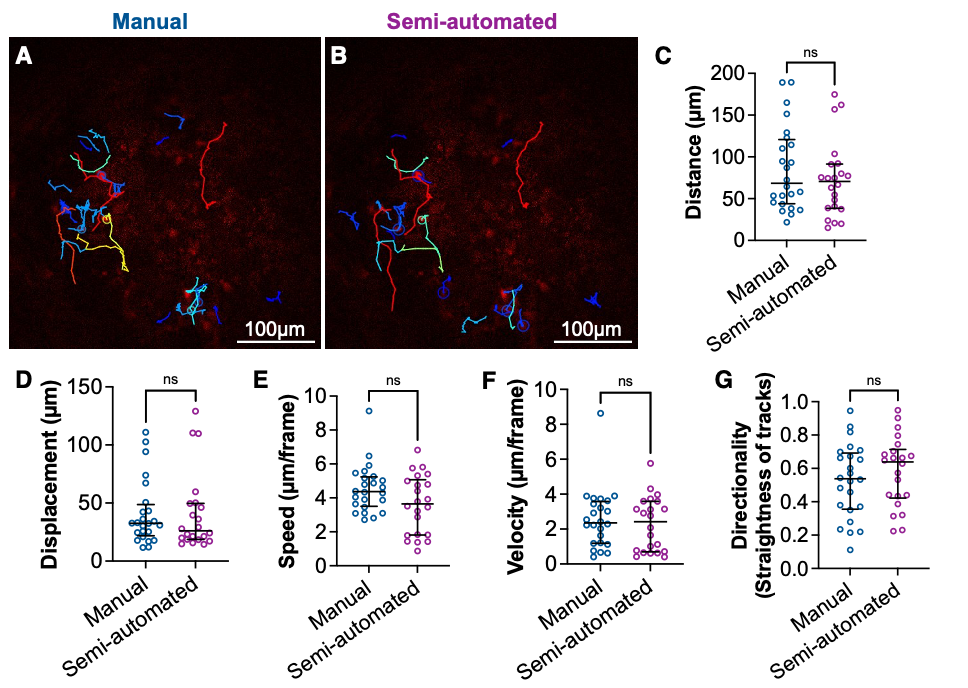
**

**S5 Fig. Comparison between manual and semi-automated tracking protocols.** An IV-MLSM timelapse video was analyzed by manual tracking after subtracting the minimum intensity projection from the stack. The same video was analyzed using the semi-automated tracking protocol. Representative 2D IV-MLSM image with overlaid tracks generated by manual tracking (A) and semi-automated tracking (B). Quantification of neutrophil distance traveled (C), displacement (D), mean speed (E), mean velocity (F), and directionality (G), was performed and the data are presented with the median and interquartile range. (ns = not significant as determined by Mann-Whitney tests (n = 24 tracks for manual tracking and 22 tracks for semi-automated tracking)).

**S1 Video.** Representative IV-MLSM timelapse of the neutrophils with the tracks overlaid proximal to a sterile pin (left) and an infected pin (right) at 2 hours. Scale bar = 100µm. Note that the undetected neutrophils have displacement lower than 16µm.

**S2 Video.** Representative IV-MLSM timelapse of the neutrophils with the tracks overlaid proximal to a sterile pin (left) and an infected pin (right) at 4 hours. Scale bar = 100µm. Note that the undetected neutrophils have displacement lower than 16µm.

**S3 Video.** Representative IV-MLSM timelapse of the neutrophils with the tracks overlaid proximal to a sterile pin (left) and an infected pin (right) at 6 hours. Scale bar = 100µm. Note that the undetected neutrophils have displacement lower than 16µm.

**S4 Video.** Representative IV-MLSM timelapse of the neutrophils (left) and USA300 MRSA (right) with the tracks overlaid proximal to an infected pin at 2 hours. Scale bar = 100µm. Note that the undetected neutrophils and bacteria have displacement lower than 16µm.

**S5 Video.** Representative IV-MLSM timelapse of the neutrophils and bacteria proximal to an infected pin at 2 hours (same experiment as Supplementary Videos 1 and 2). The insets are tracking two different bacteria. Scale bar = 100µm. Note that the undetected neutrophils and bacteria have displacement lower than 16µm.

**S6 Video.** Comparison between manual and semi-automated tracking. IV-MLSM timelapse of the neutrophils with the tracks overlaid for manual tracking (left) and semi-automated tracking (right). Scale bar = 100µm. Note that the manual tracking resulted in tracks from faintly fluorescent cells which is a source of user-variability.

**S7 Video.** Representative IV-MLSM timelapse of the neutrophils with the tracks overlaid. Note that the neutrophils in the first inset appear distinctly and then cluster until which time the cells cannot be tracked. Once the cells emerged from the cluster, we were able to track these cells. This is an inherent limitation of our and previous tracking protocols. Note that neutrophils in the second inset were not tracked since they appear dim and have lower displacement. These cells can be included for tracking by adjusting the TWS and TrackMate parameters. Scale bar = 100µm.

**S1 Table.** A comparative analysis between manual and semi-automated protocols for cell tracking. A user analyzed an IV-MLSM video either by manually tracking individual cells or by using the semi-automated protocol described in this paper. The descriptive statistics of the neutrophil kinematics as assessed by fully manual and semi-automated methods are reported here.

|  | **Distance (µm)** | | **Displacement (µm)** | | **Speed (µm/frame)** | | **Velocity (µm/frame)** | | **Directionality** | |
| --- | --- | --- | --- | --- | --- | --- | --- | --- | --- | --- |
|  | **Manual** | **Semi-automated** | **Manual** | **Semi-automated** | **Manual** | **Semi-automated** | **Manual** | **Semi-automated** | **Manual** | **Semi-automated** |
| **Minimum** | 21.7 | 15.16 | 11.75 | 14.39 | 2.71 | 0.87 | 0.40 | 0.40 | 0.111 | 0.224 |
| **25% Percentile** | 43.92 | 38.48 | 21.87 | 18.64 | 3.50 | 1.81 | 1.19 | 0.69 | 0.356 | 0.422 |
| **Median** | 68.36 | 70.55 | 32.59 | 26.03 | 4.37 | 3.65 | 2.35 | 2.42 | 0.538 | 0.639 |
| **75% Percentile** | 120.8 | 91.39 | 48.74 | 49.7 | 5.24 | 5.07 | 3.58 | 3.59 | 0.693 | 0.714 |
| **Maximum** | 189.2 | 174.7 | 110.9 | 129.1 | 9.12 | 6.82 | 8.63 | 5.77 | 0.946 | 0.949 |
| **Range** | 167.5 | 159.6 | 99.13 | 114.7 | 6.41 | 5.95 | 8.22 | 5.37 | 0.835 | 0.725 |
|  |  |  |  |  |  |  |  |  |  |  |
| **Mean** | 85.63 | 72.37 | 41.16 | 40.77 | 4.54 | 3.61 | 2.56 | 2.28 | 0.525 | 0.582 |
| **Std. Deviation** | 50.61 | 45.21 | 28.26 | 33.53 | 1.41 | 1.74 | 1.72 | 1.56 | 0.222 | 0.210 |

**S1 Appendix – ImageJ macro for pre-processing**

Open the timelapse using Fiji and run this macro to finish all the pre-processing in one step. After the macro runs, follow the protocol from step 11 to generate quantitative tracking metrics.

run("Split Channels");*//Splits the color channels if the file is a hyperstack*
close(); *//Closes the second channel. We are only interested in the first channel (neutrophils) for our example*
RawStack = getTitle();*//Stores the title of the stack to use in downstream code*
run("Image Stabilizer", "transformation=Translation maximum_pyramid_levels=1 template_update_coefficient=0.90 maximum_iterations=200 error_tolerance=0.0000001");*//Runs the Image Stabilizer plugin with default settings*
run("Z Project...", "projection=[Min Intensity]");*//Computes the minimum intensity projection of the stack*
MinProjection = getTitle();
imageCalculator('Subtract stack', RawStack, MinProjection);*//Subtracts the minimum intensity projection from the raw stack*
Processedstack = getTitle();
selectWindow(MinProjection);*//Selects the minimum intensity projection window*
close();
macroFilePath = getDirectory("current");*//Save the macro (.ijm file) and the Weka Segmentation classifier (.model file) in the same folder as the .tif file*
fileName = "NeutrophilClassifier.model";*//Replace the file name with the classifier model*
filePath = macroFilePath + fileName;
run("Trainable Weka Segmentation");*//Opens Weka segmentation plugin*
wait(1000);
call("trainableSegmentation.Weka_Segmentation.loadClassifier", filePath);*//Loads the classifier file*
wait(5000);
call("trainableSegmentation.Weka_Segmentation.getProbability");*//Generates probability maps*
wait(5000);
selectImage("Probability maps");
run("Make Subset...", "channels=2 slices=1-60");*//Our classifier has labels for both background and neutrophils. So we are extracting the neutrophil probability map.*
run("Properties...", "channels=1 slices=1 frames=60 pixel_width=0.8286412 pixel_height=0.8286412 voxel_depth=1.0000000 frame=[28.17 sec]");*//Replace the values with the image metrics*
close("Probability maps");
run('TrackMate' ,"use_gui=true " + "display_results=true " + "radius=7.0 " + "threshold=0.001 " + "subpixel=true " + "median=true " + "channel=1 ");*//Opens TrackMate*

**S2 Appendix – TWS classifier**

The following training features were used for classification in TWS. The best classifier was identified by visual comparison of the probability maps with the original stacks for segmentation accuracy. In addition, the tracks generated from the probability maps from these classifiers were compared with that of manual tracking. Classifier 1 performed better or at least as well as other classifiers and was therefore chosen for subsequent analysis.

Noise removal features | Edge detection features | Texture description features

| **Classifier 1** | **Classifier 2** | **Classifier 3** | **Classifier 4** | **Classifier 5** | **Classifier 6** |
| --- | --- | --- | --- | --- | --- |
| Gaussian blur  Sobel  Hessian  Difference of Gaussians | Gaussian blur  Sobel  Hessian  Difference of Gaussians  Laplacian | Gaussian blur  Sobel  Hessian  Difference of Gaussians  Laplacian  Median | Gaussian blur  Sobel  Hessian  Difference of Gaussians  Laplacian  Median  Bilateral | Gaussian blur  Sobel  Hessian  Difference of Gaussians  Laplacian  Median  Kuwahara | Gaussian blur  Sobel  Hessian  Difference of Gaussians  Laplacian  Median  Bilateral  Entropy |

**S3 Appendix – MATLAB code for quantification of tracks**

clear

clc

%Import excel data

Userinput=input("Input the file name" , 's');%Insert the file name here. The xlsx file has to be in the same folder as matlab code.

Data=xlsread(Userinput); %Reads the excel sheet and stores information in matrix

partid = [Data(:,9)]; %Setting array parameters partid. (This is the array that the code is based off of)

n=length(partid); %Designating a length

d=1; %Comparison variable

c=1; %Column count variable

r=1; %Row count variable

%Section the data and group into different arrays based on partid

for i=1:n

if partid(i) == d;

ID{r,c}= Data(i,(9:13));

r=r+1;

else

d=d+1;

c=c+1;

ID{1,c}=Data(i,(9:13));

r=2;

end

end

%Compile the matrix based on partid numbers

for c=1:length(ID(1,:))

iso{c} = cell2mat(ID(:,c));

end

%iso will be an matrix that separates the different particles into their

%respective arrays.

%Body of the code that computes parameters in iso array

z=length(iso);

for j = 1:z;

cell=iso{j};

p=length(cell(:,1));

Time=cell(:,2); %sets Time to the second column of 'cell' matrix

%converting the x/y position to pixel value.

for i = 1:p

x=cell(:,4);

y=cell(:,5);

xpixel(i)=x(i);

ypixel(i)=y(i);

end

%portion of the code that compiles dx,dy, euclidean, speed values

for i = 1:(p-1);

dx(i)=xpixel(i+1)-xpixel(i);

dy(i)=ypixel(i+1)-ypixel(i);

euclidean(i)=sqrt((dx(i)^2)+(dy(i)^2));

speed(i)=euclidean(i)/(Time(i+1)-Time(i));

end

meanspd=mean(speed);

totaldistance=sum(euclidean);

xdisplacement= xpixel(p)-xpixel(1);

ydisplacement= ypixel(1)-ypixel(p);

%Directionality if statement comparisons

if (xdisplacement > 0) & (ydisplacement > 0);

theta=abs(atan(ydisplacement/xdisplacement));

directionality=((theta)*(180/pi));

elseif (xdisplacement < 0) & (ydisplacement > 0);

theta=abs(atan(ydisplacement/xdisplacement));

directionality= (180-((180/pi)*theta));

elseif (xdisplacement < 0) & (ydisplacement <= 0);

theta=abs(atan(ydisplacement/xdisplacement));

directionality= (180+((180/pi)*theta));

elseif (xdisplacement > 0) & (ydisplacement <= 0);

theta=abs(atan(ydisplacement/xdisplacement));

directionality= (360-((180/pi)*theta));

elseif (xdisplacement == 0) & (ydisplacement > 0);

theta=90;

directionality = theta;

elseif (xdisplacement == 0) & (ydisplacement < 0);

theta=270;

directionality = theta;

elseif (xdisplacement == 0) & (ydisplacement ==0);

directionality = 999;

end

%Storing information in new matrix columns

iso{j}(:,6)=xpixel;

iso{j}(:,7)=ypixel;

iso{j}(2:p,8)=dx;

iso{j}(2:p,9)=dy;

iso{j}(1,10)= sqrt((ydisplacement^2)+(xdisplacement^2));

iso{j}(1,11)= (sqrt((ydisplacement^2)+(xdisplacement^2))/(p-1));

iso{j}(1,12)= totaldistance;

iso{j}(1,13)= (totaldistance/(p-1));

iso{j}(1,14)= xdisplacement;

iso{j}(1,15)= ydisplacement;

iso{j}(1,16)= directionality;

iso{j}(1,17)= xpixel(1)

iso{j}(1,18)= ypixel(p)

clear xpixel ypixel dx dy euclidean speed p directionality

end

%Combining arrays that were previously separated.

final=cat(1,iso{1:(j)});

%Array of excel titles

titles= ["partid"; "nspot"; "time";"xpos";"ypos";"xpixel";"ypixel";"dx";"dy"; "total displacement";"displacement";"total distance"; ...

"mean speed";"xdisplacement";"ydisplacement";"directionality"; "initial x position"; "initial y position"]';

%Combining array titles with matrix of data

finalt=final(:,[1:2 10:18]);

str2double(finalt);

ind= find((finalt(:,5)==0));

finalt(ind, :) = [];

titles1= ["partid"; "nspot" ; "total displacement";"velocity";"total distance"; ...

"speed";"xdisplacement";"ydisplacement";"directionality";"initial x position"; "initial y position"]';

final1=cat(1,titles1,finalt);

final=cat(1,titles,final);

%Writes the final calculations and terms into the second sheet of the excel

%file.

%Excel sheet has to have a second and third sheet for this function to work.

writematrix(final,Userinput,'Sheet',2);

writematrix(final1,Userinput,'Sheet',3);

**S4 Appendix – MATLAB code for quantification of parallel migration pattern**

%% CMSR Cell Tracking Contact Time

clear

clc

%Read data that has been processed using data analysis code.

Data=readmatrix('Copy of C1-Mouse 95, 6hr_Tracks.xlsx', 'Sheet', 'Sheet2');

Data1=readmatrix('Copy of C2-Mouse95,6hr_Tracks.xlsx', 'Sheet', 'Sheet2');

%Subset data to recognize the particle IDs within track data

partid = [Data(2:end,1)];

partid1 = [Data1(2:end,1)];

d=1; %Comparison variable

c=1; %Column count variable

r=1; %Row count variable

%Identify matrix length

n=length(partid);

for i=1:n %Matrix formatting. This is to determine which data parts we need.

if partid(i) == d;

ID{r,c}= Data(i,(1:5));

r=r+1;

else

d=d+1;

c=c+1;

ID{1,c}=Data(i,(1:5));

r=2;

end

end

%Imbedding the matrix within a matrix(All the required information we need

%for each particle.

for c=1:length(ID(1,:))

iso{c} = cell2mat(ID(:,c));

end

j=length(partid1);

d1=1; %Comparison variable

c1=1; %Column count variable

r1=1; %Row count variable

%Similar process but for the tracks for the bacteria.

for i=1:j

if partid1(i) == d1;

ID1{r1,c1}= Data1(i,(1:5));

r1=r1+1;

else

d1=d1+1;

c1=c1+1;

ID1{1,c1}=Data1(i,(1:5));

r1=2;

end

end

%Imbedding the matrix information of each bacteria into a new matrix

for c1=1:length(ID1(1,:))

iso1{c1} = cell2mat(ID1(:,c1));

end

%Identifying the length of both matrix groups.

%This is important because we need this information for comparison

p=length(iso);

n=length(iso1);

%Bulk of the for loop. It goes through the matrix information and compares

%the different x y coordinates between the two data files to access contact

%time. If the centroids of cells are within 16 microns of one another, they

%count that as one contact. This code will output the total contact frames

%and with which bacterial cell.

for m = 1:p;

cell=iso{m};

xy=cell(:,4:5);

time= cell(:,3);

for i= 1:n;

cell1=iso1{i};

xy1=cell1(:,4:5);

time1= cell1(:,3);

o=length(xy);

l=length(xy1);

count=0;

for k= 1:o;

comp=[xy(k,1),xy(k,2)];

comptime=time(k);

for c = 1:l;

comp1=[xy1(c,1),xy1(c,2)];

comptime1=time1(c);

if abs(comptime-comptime1) == 0;

dy=(xy1(c,2))-(xy(k,2));

dx=(xy1(c,1))-(xy(k,1));

if sqrt((dy^2)+(dx^2)) <= 16;

count= count + 1;

else

count=count;

end

end

end

if count ~=0;

iso{m}(c,6)=iso1{i}(1,1);

iso{m}(c,7)=count;

end

end

end

end

%This portion is the clean up the output for most relavent information.

%Eliminates the 0s that are outputted in the matrix as a result of the loop

z=1;

v=length(iso);

for m = 1:v ;

cell2=iso{m};

if width(cell2)==7;

isosub{1,m}= cell2(:,[1 6 7]);

else

end

end

%Isosub is the subset of the matrix that allows us to look at the particle

%in contact with (first column) and the (contact time)

isosub=isosub(~cellfun('isempty',isosub));

v=width(isosub);

for m= 1:v;

cell2=isosub{m};

ind=find(cell2(:,2)==0);

pd=([cell2(1,1) 0]);

cell2(ind,:)=[];

isosub{m}=cat(2,pd,cell2);

end

%Concatenates the data and titles

final=cat(1, isosub{1:v});

final1=final(:, [1 4 5]);

titles= ["Original Neutrophil particle ID", "Bacterial ParticleID in contact with", "Timeframes in Contact"];

final1=cat(1, titles, final1);

%Write the information to a new sheet of the neutrophil data file.

writematrix(final1, 'Copy of C1-Mouse 95, 6hr_Tracks.xlsx', 'Sheet', 'Sheet4');
