# Supplementary material for: A semi-automated cell tracking protocol for quantitative analyses of neutrophil swarming to sterile and S. aureus contaminated bone implants in a mouse femur model
Source: PLoS One. 2024 Jun 20;19(6):e0296140. doi: 10.1371/journal.pone.0296140 (PMC11189170; doi:10.1371/journal.pone.0296140)
Supplement: Attachment — Submitted filename: Response to Reviewers.docx [file pone.0296140.s009.docx]

Journal requirements:

1. When submitting your revision, we need you to address these additional requirements.

Please ensure that your manuscript meets PLOS ONE's style requirements, including those for file naming. The PLOS ONE style templates can be found at 
[https://journals.plos.org/plosone/s/file?id=wjVg/PLOSOne_formatting_sample_main_body.pdf](https://urldefense.com/v3/__https:/journals.plos.org/plosone/s/file?id=wjVg*PLOSOne_formatting_sample_main_body.pdf__;Lw!!CGUSO5OYRnA7CQ!ZSsereCjBIF0VOM-YZtvonBCtzOhdgh_fhvidLkN9YGBdDa8G6JeOVRkufHN_xbSsDiHU9EMu-_GfpA1FnLdRdI$)

and 
[https://journals.plos.org/plosone/s/file?id=ba62/PLOSOne_formatting_sample_title_authors_affiliations.pdf](https://urldefense.com/v3/__https:/journals.plos.org/plosone/s/file?id=ba62*PLOSOne_formatting_sample_title_authors_affiliations.pdf__;Lw!!CGUSO5OYRnA7CQ!ZSsereCjBIF0VOM-YZtvonBCtzOhdgh_fhvidLkN9YGBdDa8G6JeOVRkufHN_xbSsDiHU9EMu-_GfpA1kvABM6I$).

As suggested, we formatted the file names according to PLOS ONE’s style requirements.

2. Please note that PLOS ONE has specific guidelines on code sharing for submissions in which author-generated code underpins the findings in the manuscript. In these cases, all author-generated code must be made available without restrictions upon publication of the work. Please review our guidelines at [https://journals.plos.org/plosone/s/materials-and-software-sharing#loc-sharing-code](https://urldefense.com/v3/__https:/journals.plos.org/plosone/s/materials-and-software-sharing*loc-sharing-code__;Iw!!CGUSO5OYRnA7CQ!ZSsereCjBIF0VOM-YZtvonBCtzOhdgh_fhvidLkN9YGBdDa8G6JeOVRkufHN_xbSsDiHU9EMu-_GfpA14bfA6Cc$) and ensure that your code is shared in a way that follows best practice and facilitates reproducibility and reuse.

As suggested, all the codes used for generating the results in the manuscript are included in the Appendix of the supplementary materials. We also uploaded the MATLAB codes to a GitHub repository and the DOI is now included in the data availability statement.

“**Data Availability Statement:** All relevant data are within the manuscript and its Supporting Information files. The MATLAB codes used for this study are available on GitHub: Lee, K., Lekkala, S., & Yeh, S. A. Quantification of tracks generated from TrackMate [Computer software]. <https://github.com/SashankLekkala/Semi-automated-cell-tracking>. DOI: 10.5281/zenodo.10814082.”

3. To comply with PLOS ONE submissions requirements, in your Methods section, please provide additional information regarding the experiments involving animals and ensure you have included details on (1) methods of sacrifice, (2) methods of anesthesia and/or analgesia, and (3) efforts to alleviate suffering.

We added the requested details to the Methods section. Please refer to our response to Reviewer 1, comment 5.

5. Thank you for stating the following financial disclosure: 
 [Part of this work was supported by the AOTrauma Clinical Priority Program, and the National Institutes of Health: R21 AR081050 (C. Xie), P30 AR069655 (E. Schwarz) & P50 AR072000 (E.Schwarz).].  
Please state what role the funders took in the study.  If the funders had no role, please state: ""The funders had no role in study design, data collection and analysis, decision to publish, or preparation of the manuscript."" 
If this statement is not correct you must amend it as needed. 
Please include this amended Role of Funder statement in your cover letter; we will change the online submission form on your behalf.

As suggested, we added the funder role statement:

“**Funding sources:** Part of this work was supported by the AOTrauma Clinical Priority Program, and the National Institutes of Health: R21 AR081050 (C. Xie), P30 AR069655 (E. Schwarz) & P50 AR072000 (E. Schwarz). The funders had no role in study design, data collection and analysis, decision to publish, or preparation of the manuscript.”

6. Please include the reference section of your manuscript.

The references are included in the manuscript after the Acknowledgments.

7. We note that Figure(s), 1A, B, C, D,E, F, 2A,B, 3A, 4A, S1A,B, S2A and B in your submission contain copyrighted images. All PLOS content is published under the Creative Commons Attribution License (CC BY 4.0), which means that the manuscript, images, and Supporting Information files will be freely available online, and any third party is permitted to access, download, copy, distribute, and use these materials in any way, even commercially, with proper attribution. For more information, see our copyright guidelines: [http://journals.plos.org/plosone/s/licenses-and-copyright](https://urldefense.com/v3/__http:/journals.plos.org/plosone/s/licenses-and-copyright__;!!CGUSO5OYRnA7CQ!ZSsereCjBIF0VOM-YZtvonBCtzOhdgh_fhvidLkN9YGBdDa8G6JeOVRkufHN_xbSsDiHU9EMu-_GfpA1oSJFxXs$).
We require you to either (1) present written permission from the copyright holder to publish these figures specifically under the CC BY 4.0 license, or (2) remove the figures from your submission:
a. You may seek permission from the original copyright holder of Figure(s), 1A, B, C, D,E, F, 2A,B, 3A, 4A, S1A,B, S2A and B to publish the content specifically under the CC BY 4.0 license. 
We recommend that you contact the original copyright holder with the Content Permission Form ([http://journals.plos.org/plosone/s/file?id=7c09/content-permission-form.pdf](https://urldefense.com/v3/__http:/journals.plos.org/plosone/s/file?id=7c09*content-permission-form.pdf__;Lw!!CGUSO5OYRnA7CQ!ZSsereCjBIF0VOM-YZtvonBCtzOhdgh_fhvidLkN9YGBdDa8G6JeOVRkufHN_xbSsDiHU9EMu-_GfpA1e72kYIo$)) and the following text:
“I request permission for the open-access journal PLOS ONE to publish XXX under the Creative Commons Attribution License (CCAL) CC BY 4.0 ([http://creativecommons.org/licenses/by/4.0/](https://urldefense.com/v3/__http:/creativecommons.org/licenses/by/4.0/__;!!CGUSO5OYRnA7CQ!ZSsereCjBIF0VOM-YZtvonBCtzOhdgh_fhvidLkN9YGBdDa8G6JeOVRkufHN_xbSsDiHU9EMu-_GfpA1fFKN0pI$)). Please be aware that this license allows unrestricted use and distribution, even commercially, by third parties. Please reply and provide explicit written permission to publish XXX under a CC BY license and complete the attached form.”
Please upload the completed Content Permission Form or other proof of granted permissions as an ""Other"" file with your submission.  
In the figure caption of the copyrighted figure, please include the following text: “Reprinted from [ref] under a CC BY license, with permission from [name of publisher], original copyright [original copyright year].”
b. If you are unable to obtain permission from the original copyright holder to publish these figures under the CC BY 4.0 license or if the copyright holder’s requirements are incompatible with the CC BY 4.0 license, please either i) remove the figure or ii) supply a replacement figure that complies with the CC BY 4.0 license. Please check copyright information on all replacement figures and update the figure caption with source information. If applicable, please specify in the figure caption text when a figure is similar but not identical to the original image and is therefore for illustrative purposes only.

The authors created the images/videos in the Figures and Supporting Information files and the images/videos have not been previously copyrighted.

We are most grateful to the Reviewers for their helpful comments, which we address below. The Reviewers’ queries are numbered and repeated in black, bold, and italicized text, and our responses follow in blue text. New text in the manuscript is indicated in blue text.

**Reviewer #1:** The manuscript titled "A Semi-Automated Cell Tracking Protocol for Quantitative Analyses of Neutrophil Swarming to Sterile and *S. aureus* Contaminated Bone Implants in a Mouse Femur Model" presents a well-structured protocol for accurate and reproducible cell tracking in IV-MLSM videos. The semiautomated tracking protocol utilizing trainable Weka segmentation (TWS) and TrackMate effectively minimizes user intervention and offers an efficient method for tracking individual cells in diverse experimental scenarios. Nevertheless, to further enhance the manuscript's quality, I recommend the authors to address certain key points:

***1. In the Results section, the "Parallel Migration Pattern of Neutrophils and Bacteria" is intriguing. I think a more in-depth analysis of cell interactions would significantly enrich the understanding of this phenomenon.***

We thank the Reviewer for the praise of our work and suggestions to improve the manuscript. We are currently planning dedicated biological studies that may manipulate chemotactic behaviors to further analyze this phenomenon. This part of the experiment however requires further optimization on the biology side (e.g. imaging time point, treatment dosage, etc) and is beyond the scope of this manuscript that focuses on the development of image analysis protocols. We appreciate that the Reviewer agreed on the value of parallel migration pattern, thus we have added the following paragraph to the Discussion section to elaborate on the potential applications (pages 17, lines 360-367):

“Several studies performed longitudinal imaging of neutrophils and macrophages and reported chemotaxis followed by phagocytosis but did not fully quantify this behavior.(1,2) In this study, we proposed a quantitative method to study this parallel migration pattern as an indicator of chemotactic behaviors. Using cell tracking data, we demonstrated that we could determine the number of contacting cell pairs and their contact time. Our future goal is to implement this algorithm, especially in the context of different antibiotic treatment regimens to understand the correlation between parallel migration, phagocytosis, and the therapeutic outcome.”

***2. The Discussion section mentions that "some of the cells were not tracked by the protocol." It would be beneficial for the authors to explore and clarify the reasons behind this, assessing whether it introduces any bias in the interpretation of the results.***

We thank the Reviewer for pointing this out, and we have clarified this further in the Discussion section (pages 14-15, lines 303-308 and 319-326), regarding the two scenarios when the cells are not tracked and the associated impact on data interpretation.

“For the analysis, cells with low fluorescence intensity and/or low displacement are excluded. This exclusion is not a limitation but rather a conscious decision for our application. We excluded dimly fluorescent cells to exclude autofluorescent cells that are not neutrophils. These low-intensity cells can be easily included in the analysis by changing the TWS classifier to be more conservative. Similarly, we intentionally excluded cells with smaller displacement (<16µm) which can be included by changing the displacement filter in TrackMate.”

“However, the limitation of the protocol is that when the cells are crowded, the neutrophils cannot be distinguished from one another (S7 Video, Inset 1). This distinction is not possible even manually and would require other tools such as cell membrane labeling to distinguish crowded cells separately. In these cases, the cells that merged as a cluster may be misidentified as one cell, which needs to be edited manually. For such cases, we opted to end the tracks when the cells cluster, and then restart tracking after the cells re-emerged from the cluster. This edit shortens the tracks resulting in lower distance and displacement for these cells. In contrast, time-normalized metrics such as speed and velocity are less susceptible to this limitation.”

**S7 Video.** Representative IV-MLSM timelapse of the neutrophils with the tracks overlaid. Note that the neutrophils in Inset 1 appear distinctly and then cluster until which time the cells cannot be tracked. Once the cells emerged from the cluster, new tracks were started for these cells. This limitation is inherent in our and previous tracking protocols. The neutrophils in the Inset 2 were excluded because they were out of focus and had lower displacement (<16µm). These cells can be included for tracking by adjusting the TWS and TrackMate parameters. Scale bar = 100µm.

***3. To bolster the reliability of the protocol, a comparative analysis is advised. Comparing the results of manual analysis performed by a group of users with those obtained from the same IV-MLSM video using the semiautomated protocol would provide valuable insights.***

As suggested by the Reviewer, we performed a comparative analysis between manual and semi-automated tracking and discussed the results in the Discussion section. (pages 14-15, lines 309-318)

“We performed a comparative analysis between the results from manual and semi-automated tracking protocols (S5 Fig, S6 Video). We performed manual tracking using TrackMate after performing minimum intensity projection subtraction where a user tracked individual cells (S5 Fig A). Then we compared the results with those from the semi-automated tracking protocol (S5 Fig B). Most of the tracks were similar between both cases (S5 Fig, S1 Table). However, as expected, manual tracking resulted in tracks from faintly fluorescent cells which are potentially auto-fluorescent cells that are not neutrophils (also a source of user variability) (S6 Video). In addition, we showed in S2 Fig that manual tracking suffers from inter-person variability. This limitation is overcome by the semi-automated protocol, as TWS segments the cells of interest.”

**S5 Fig. Comparison between manual and semi-automated tracking protocols.** An IV-MLSM timelapse video was analyzed by manual tracking after subtracting the minimum intensity projection from the stack. The same video was analyzed using the semi-automated tracking protocol. A representative 2D IV-MLSM image with overlaid tracks generated by manual tracking is shown in (A) and semi-automated tracking is shown in (B). Quantification of neutrophil distance traveled (C), displacement (D), mean speed (E), mean velocity (F), and directionality (G), was performed, and the data are presented with the median and interquartile range. (ns = not significant as determined by Mann-Whitney tests (n = 24 tracks for manual tracking and 22 tracks for semi-automated tracking)).

**S6 Video. Comparison between manual and semi-automated tracking.** IV-MLSM timelapse of the neutrophils with the tracks overlaid for manual tracking (left) and semi-automated tracking (right). Scale bar = 100µm. Note that the manual tracking resulted in tracks from faintly fluorescent cells which is a source of user-variability.

***4. In the Methods section, under "MRSA Strain and Implants," a comprehensive description of the process and conditions is needed.***

We thank the Reviewer for pointing this out. As suggested, we have added additional details to the methods (page 5, lines 93-106):

“The most prevalent community-acquired methicillin-resistant *Staphylococcus aureus* (MRSA) strain, USA300, was used for all experiments. The bacteria were grown on tryptic soy agar (TSA) or in tryptic soy broth (TSB) at 37°C. We transformed USA300 LAC (ATCC AH1680) with the pCM29-*sarA*::*ecfp* or pCM29-*sarA*::*egfp* reporter plasmids to generate ECFP and EGFP expressing USA300 strains, respectively, as we previously reported.(3) Positive pCM29 plasmid transformation renders USA300 LAC resistant to chloramphenicol. Therefore, ECFP^+^ and EGFP^+^ USA300 LAC transformants were positively selected in TSB with 10 μg/mL chloramphenicol. Subsequently, fluorescence microscopy was used to confirm the positive transformation of ECFP and EGFP into the USA300 LAC strain.(4)

A flat titanium wire (cross-section 0.2 mm × 0.5 mm; MicroDyne Technologies, Plainville, CT) was cut to 4 mm length and bent into an L-shaped implant: long side 3 mm, short side 1 mm.(5) After sterilization, the implant was incubated in the overnight culture of transformed USA300 for 30 minutes prior to the implantation procedure as previously described.(4)”

***5. In the "Animal Surgery and LIMB" subsection, it is crucial to specify the mouse strain used and detail the rearing conditions.***

We have added additional details to the methods to enhance the clarity of the mouse model (pages 5-6, lines 108-122):

“All animal research was performed under protocols approved by the University of Rochester Committee on Animal Resources (UCAR-2019-015). We obtained Catchup mice (C57BL/6 genetic background) (6) from Dr. Minsoo Kim (University of Rochester Medical Center) and maintained the colony. For this study, we used both male and female mice that were 12-20 weeks of age. Thirty minutes before surgery, the mice were given Buprenorphine SR (1 mg/kg) subcutaneously. The mice were then anesthetized with xylazine (10 mg/kg) and ketamine (100 mg/kg) administered intraperitoneally. The surgical procedure and the imaging setup were based on protocols previously described.(4) Briefly, the right femur was implanted with a customized LIMB system. The implant enabled imaging of a fixed region of interest (ROI) proximal to an L-shaped transcortical pin in the diaphyseal bone marrow. The mice received either a sterile pin or a USA300 contaminated pin.

The mice were imaged at 2-, 4-, and 6-hours post-implantation while anesthetized. During imaging, the mice were lying prone, and the LIMB implant was connected to a custom adaptor for proper placement of the objective lens to find the same ROI. The animals were euthanized by CO_2_ overdose followed by cervical dislocation to ensure euthanasia.”

***6. Regarding Figure 2, does "n=25-57 tracks" introduce potential statistical bias? An elaboration on this aspect would be beneficial.***

We thank the Reviewer for raising this question. We would like to clarify that in Figure 2, the differences in the number of tracks (n) are from different mice but not from different users (For mice 1, 2, and 3, User 1 computed 36, 25, and 56 tracks while User 2 computed 40, 25, and 57 tracks respectively). As the data does not follow a normal distribution, we then employed a non-parametric statistical test. In addition, because we observed substantial data spread (high inter-quartile range), which may result in statistical insignificance for small n, we calculated the interclass correlation coefficients (ICC). For a given parameter, ICC takes into account the variability for each individual track giving an accurate estimate of user variability. We calculated ICC to quantify variability in manual tracking and the values ranged from 0.85-0.99 which indicates modest inter-person agreement. We have added ICC for manual tracking to the Results section.

“The major limitation of manual tracking is high inter-user variability. The two users assigned to analyze the same IV-MLSM video often identified different tracks. The lack of consistency is shown in the data distribution, where the median displacement and velocity of neutrophils differed by 25% and 22% respectively between the two users (S2 Fig). While these results were not statistically significant, such substantial numerical differences impede reliable conclusions. Furthermore, the inter-class correlation coefficient for manual tracking was as low as 0.85, indicating only a modest user agreement.”

***7. The legend for Figure 2 should include detailed explanations of terms such as "ns" to improve reader comprehension.***

We thank the Reviewer for pointing this out. To address this, we added the clarification (“ns = not significant”) to the figure legends.

**“Fig 2. The tracking protocol resulted in low inter-person variability in generating tracks.** Two users independently analyzed three IV-MLSM timelapse videos and the generated track parameters were compared. Representative 2D IV-MLSM image with overlaid tracks generated by user 1 (A) and user 2 (B). The differences in the tracks generated by both users are shown with white arrows. Semiautomated quantification of neutrophil distance traveled (C), displacement (D), mean speed (E), mean velocity (F), and directionality (G), was performed and the data are presented with the median and interquartile range. (ns = not significant as determined by Mann-Whitney tests adjusted by the Holm-Šídák method (n=25-57 tracks. Representative images from N=3 mice)).”

***8. The statistical results in Figure 3B need a more thorough description to enhance clarity and understanding.***

We thank the Reviewer for pointing this out. As suggested, we added the statistical details to the figure 3B.

**“Fig 3. Increased neutrophil swarming proximal to MRSA contaminated vs. sterile bone implant.** *Catchup* mice were challenged with a sterile or MRSA-contaminated transfemoral implant and neutrophil swarming behaviors were quantified from 30min IV-MLSM videos obtained at the indicated time post-implantation. (A) Representative 2D IV-MLSM images with overlaid neutrophil tracks proximal to sterile and infected pin at 2-, 4-, and 6-hours post-implantation (S1-3 Videos). (B) Change in neutrophil volume with time proximal to infected and sterile implants calculated using Imaris (S1 Fig). Data is shown as mean ± standard deviation. Unpaired t-tests were used to test the differences between infected and sterile conditions (n=3). Semiautomated quantification of neutrophil distance traveled (C), displacement (D), mean speed (E), mean velocity (F), and directionality (G), was performed and the data are presented with the median and interquartile range. (*p < 0.05 as determined by Mann-Whitney tests adjusted by the Holm-Šídák method (n=105-316 tracks/group/timepoint, N=3 mice/group)).”

We also clarified in the Results section that we did not observe statistical differences in neutrophil volumes between infected and sterile conditions.

“Consistent with the infection status, the neutrophil volume proximal to the pin increased with time in the infected animals but remained consistent in uninfected animals (Fig 3A-B, S1-3 Videos). However, these results were not statistically significant.”

To improve the understanding of these results in Figure 3, we expanded our discussion. (pages 15-16, lines 332-340)

“Using the LIMB system, we studied neutrophil behavior in response to sterile or MRSA-contaminated implants. As expected, the neutrophil volume proximal to the infected pin was higher compared to that of the sterile pin indicating an active response to infection. Furthermore, in the infected mice, the neutrophils traveled farther (higher distance and displacement) and faster (higher speed and velocity) especially at later time points potentially indicating more potent activation and chemotactic signals compared to the uninfected mice. Future studies with a focus on the quantification of these signals will help elucidate the mechanisms of neutrophil activation and migration. Then the tracking metrics can be validated against these biological outcomes.”

***9. On line 279, "error-prone regions (e.g., cells with small displacement, low intensity, or located at the boundaries of the field of view)" are mentioned. Given these limitations, it is crucial to discuss how the reliability of the results is assured through user verification and the protocol's capabilities.***

The Reviewer raised a valid concern. To address this, we included additional descriptions in the Discussion (please refer to the response to comment 2). Specifically, these error-prone regions are corrected manually by the user (Figure 1A, 1E, 1F). To verify if correcting these incorrect tracks results in user bias, we tested the reproducibility of our protocol. As reported in Figure 2, despite these error-prone regions, our protocol remains highly reproducible. Furthermore, we added an additional case to prove reproducibility across different disease states as requested by Reviewer 2 (please refer to the response to Reviewer 2, comment 19).

***10. While the protocol focuses on tracking, it would be interesting to see if it can be extended to analyze cell behavior, such as changes in cell morphology or interactions with other cells.***

We thank the Reviewer for bringing up these interesting perspectives. We anticipate that the protocol can be extended to study cellular interactions, for example, by using the parallel migration pattern metrics we described in Figure 4. These metrics can be applied to longitudinally and quantitatively correlate cell chemotactic behaviors and phagocytosis under various biological assays (such as comparing treatment regimens and infection burden).

We acknowledge that morphological alteration also provides functional information on neutrophils(7) and would be an interesting aspect to interrogate. However, the GRIN lens-based system with a small numerical aperture does not provide sufficient image resolution to visualize cell processes and fine morphological changes. Nevertheless, this analysis can be performed with in vitro studies. Upon identifying individual cells using the tracking protocol, morphological changes of the cells can be monitored using inbuilt tools in ImageJ such as “Analyze particles” with metrics like roundness and major diameter, etc. We have added morphological analyses as a potential application and discussed image resolution as a limitation in the Discussion (page 17, lines 360-371).

“Several studies performed longitudinal imaging of neutrophils and macrophages and reported chemotaxis followed by phagocytosis but did not fully quantify this behavior.(1,2) In this study, we proposed a quantitative method to study this parallel migration pattern as an indicator of chemotactic behaviors. Using cell tracking data, we demonstrated that we could determine the number of contacting cell pairs and their contact time. Our future goal is to implement this algorithm, especially in the context of different antibiotic treatment regimens to understand the correlation between parallel migration, phagocytosis, and the therapeutic outcome. Further, morphological alteration has been shown to provide functional information on neutrophils(7), and would be an interesting aspect to interrogate. Although the morphology of the identified cells can be studied using ImageJ tools such as “Analyze particles” or “MorphoLibJ”(8), the GRIN lens-based system with a small numerical aperture does not provide sufficient image resolution to visualize cell processes and fine morphological changes.”

***11. The manuscript mentions not extensively denoising images before segmentation. However, a more detailed rationale behind this choice and its impact on the results would add clarity.***

We thank the Reviewer for this suggestion and added the following description to clarify the rationale (page 13, lines 269-281). The rationale behind this choice was to maintain the morphological and texture features as much as possible. Adding extensive denoising steps increases the complexity of the protocol and may introduce user variability and/or denoising artifacts. Furthermore, one of the key advantages of TWS is the ability to train the system to detect the foreground even from a noisy background.

“We opted not to extensively denoise the image prior to segmentation to retain as many morphological and textural features as possible. Such information is a critical “training set” to identify the background in the machine learning algorithms. We subtracted the minimum intensity projection from the stack to remove any stationary artifacts. However, this method will also remove any stationary cells and the nidus. Given our focus on tracking only the moving cells, this pre-processing method proved most effective for our purpose. Adding extensive denoising steps increases the complexity of the protocol and may introduce user variability and/or denoising artifacts. This protocol can be adapted with alternative denoising techniques such as morphological filtering (for example, white top-hat) to preserve the stationary details.(20) One of the key advantages of TWS is the ability to train the system to detect the foreground even from a noisy background where the features of the background were also extracted for training. For this reason, it was shown to be the most efficient when images are directly segmented using TWS without any pre-processing.”

***12. What are the advantages of using machine analysis methods such as TWS and TrackMate for cell detection and tracking compared to conventional methods? Furthermore, what is the significance of the newly developed machine computation methods? A detailed discussion on these aspects is recommended.***

Machine learning techniques such as TWS eliminate the need for user interference. Further, the most common method to segment cells is using a global or local threshold which may not work for intravital imaging due to inherent differences in the fluorescence level of cells and substantial artifacts present in the background. TWS, on the other hand, does pixel-based segmentation which results in highly accurate segmentation, especially in the cases of intravital imaging. This is described in the original manuscript (page 12, lines 262-266).

“Further, utilizing a singular global threshold might not yield optimal results for neutrophil segmentation due to the inherent variability and low signal to noise ratios in fluorescence associated with 3D intravital imaging. TWS overcomes this limitation by using a collection of machine learning algorithms to produce pixel-based segmentation.”

TrackMate is an open-source, user-friendly plugin that allows interfacing with several segmentation techniques such as TWS, StarDist, etc. In addition, TrackMate also has several particle tracking algorithms inbuilt such as linear assignment problem, intersection-over-union, etc. (Supplementary Table 1 of (9) for detailed comparison). This is described in the original manuscript (page 4 and lines 70-81).

“To quantify neutrophil swarming kinetics, TrackMate(5,6), an open-source, user-friendly plugin available within Fiji(7) is an attractive option that allows interactive cell tracking. Previous studies have reported diverse approaches to counting and tracking cells using TrackMate.(8–12) However, some of these tracking approaches utilize computationally expensive models or are restricted to specific operating systems.(12) The most apparent cellular architecture in the bone infection setting is the accumulation of bacteria and host immune cells over time. In this regard, these protocols are sub-optimal in segmenting and tracking densely populated cells with variable shapes and fluorescence intensities which became even more problematic in the context of three-dimensional intravital multiphoton laser scanning microscopy (IV-MLSM). While manual tracking options are available,(6,12) they are subject to significant inter-observer variation. Subsequent statistical analyses are therefore not meaningful given such inter-user variability.”

We also extended the discussion on alternate segmentation and tracking software (pages 13-14, lines 288-292 and page 16, lines 351-359):

“Deep learning programs such as StarDist which also use object boundaries for segmentation may overcome this limitation with a custom training set.(10,21) However, StarDist is most effective for cases of nuclear staining or cells with star-convex shapes which may not always apply to varying shapes of patrolling cells such as neutrophils.(21)”

“Several alternatives exist for segmentation and tracking. However, the key advantage of TWS and TrackMate is their ready availability as ImageJ plugins and the user-friendly interface. TWS has been successfully used for a wide range of applications and can be an attractive tool to analyze histology sections in the future.(10,11) Machine and deep learning segmentation techniques such as StarDist, Cellpose, Ilastik, etc. are available, but they require a robust training set and classifier that needs to be generated outside of ImageJ.(12,13) The trained classifier can be called into ImageJ to segment other similar images while additional integration of the workflow will be needed. These programs may offer better integration with Python or other coding platforms if ImageJ is not required for further analysis."

**Reviewer #2:** This manuscript presents a novel protocol for retinal intravital microscopy, enabling direct observation of the effects of fluorescent *Staphylococcus aureus* on contaminated transfemoral implants and host cells in mice. By utilizing two-photon laser scanning microscopy, this approach allows direct visualization of bacterial colonization of implants and host cell responses. To quantify this process, the authors developed a semiautomated machine learning model to segment, track, and analyze the dynamics of neutrophil cells, quantifying their motility characteristics. The use of the trainable Weka Segmentation (TWS) machine learning tool for detecting and tracking individual cells among various cell types represents an improvement over previous cell tracking methods, particularly in segmenting and tracking cells of diverse shapes and fluorescence intensities. This advancement resulted in less variability and greater statistical significance in subsequent analyses. While the manuscript is concise, logical, and well-structured, there are areas that require further elaboration and improvement:

***13. A detailed explanation of the origin and the basis for the application analysis of the TWS machine learning tool developed in the study is needed.***

We thank the Reviewer for the praise of our work and suggestions to improve the manuscript. To address this point, we have provided the details pertinent to the basis of the TWS classifier in the supplementary material (S2 Appendix). To enhance the clarity, we referred to S2 Appendix in the discussion (page 7, line 145).

***14. The manuscript should specify the activity state, detection area, and orientation of the mice during fluorescence detection using the two-photon laser scanning microscope.***

We apologize for the insufficient clarity in the methods. To address this, we have included detailed methodology in the revised manuscript (pages 5-6, lines 108-122), as also described in the response to Reviewer 1, comment 5.

***15. The article mentions that individual differences can impact research results, yet the manuscript lacks detailed information on the conditions of the mice. This should be addressed.***

We have included information on the conditions of the mice in the revised manuscript to ensure the reproducibility of the results (pages 5-6, lines 108-122), as also in part described in the response to Reviewer 1, comment 5.

***16. The possibility of excessive clustering in the study of cell and bacteria interactions raises the question of how individual neutrophils are located and detected. This aspect needs clarification.***

We thank the Reviewer for bringing this up and have extended discussion on this point, as in the response to Reviewer 1’s comment 2.

As robust detection of individual crowded cells remains a major limitation in objective detection by machines or human eyes, we chose early time points after infection to avoid excessive clustering. The clustering is minimal at these time points (S1-3 Video). In the cases where we observed clustering, we ended the tracks and restarted tracking when these cells re-emerged from the cluster. We clarified this criterion in the step-by-step protocol in the supplementary material (shown below and in pages 5-6, lines 118-120 of the supplementary material). Please refer to the response to Reviewer 1, comment 2. Of note, this may result in shorter tracks (reduced displacement and distance) but is not expected to impact velocity and speed.

Analyses in the early time point are biologically meaningful to understand the mechanisms governing bacterial colonization and key defensive responses from the host.

“23. Connecting two tracks: Follow the criteria in the order below to determine if two tracks need to be connected.

a. Frame gap: If the second track begins near the end of the first track both spatially (<20µm radius) and temporally (single frame gap), then the two tracks can be connected.

b. Intensity penalty: If there are a few potential candidates from the first criterion, to decide which tracks to connect, the user can penalize changes in intensity. That is, if the neutrophils in the two tracks have similar “brightness”, they can be connected.

c. Direction penalty: Abrupt changes in the directionality can also be penalized. This penalty needs to be carefully implemented since in some cases, the neutrophils may not have a preferred direction of motion.

d. Shape/size penalty: Changes in the shape or size of the neutrophil can also be penalized.

e. In the cases where neutrophils cluster and cannot be individually segmented, end the existing tracks and start new tracks when the cells emerge from the cluster. Tracks can be manually connected as needed.”

***17. For Figure G, it is advised to maintain font size consistency with the rest of the figures.***

We thank the Reviewer for pointing out this inconsistency. To address this, we fixed the font size to match the rest of the panels.

***18. Technical Specifications and Settings: More detailed information on the technical specifications and settings used during the imaging and analysis process would be beneficial. This should include details on the parameters of the machine learning algorithms, image acquisition settings, and any preprocessing steps taken.***

We apologize for the confusion. Supplementary methods and appendices include all the step-by-step protocols of the preprocessing and TWS classifier. To enhance the clarity, we have added “see Supplementary Methods and Appendix” as needed in the Methods section (page 6, line 134 and page 7, line 145).

We have also added the filter details for EGFP to the methods where the rest of the image acquisition parameters are listed (page 6, lines 127-128).

“IV-MLSM was performed using an Olympus FVMPE-RS multiphoton imaging system (Olympus), equipped with MaiTai and InsightX3 Titanium:Sapphire lasers (Spectra-Physics, Santa Clara, CA), and an LUCPLFLN 20× (NA 0.45) air objective (Olympus, Tokyo, Japan). The lasers were tuned to 1050 nm and 860 nm and the fluorescence of ECFP/EGFP, and tdTomato were collected with 460−500 nm/495–540 nm, and 575−630 nm filters, respectively. Images were acquired at a size of 512 × 512 pixels with a 0.01-μs pixel dwell time. At 2, 4, and 6 hours after implantation, three-dimensional (424.26×424.26×60 µm^3^), time-lapse (30-second interval for 30 minutes) image stacks were acquired using FLUOVIEW (31S-SW, Olympus).”

***19. Reproducibility in Different Experimental Settings: The manuscript would benefit from additional information on the protocol’s reproducibility under various experimental conditions, such as different types of tissues or varying levels of inflammation.***

We thank the Reviewer for the comment. Due to the restriction of GRIN lens based LIMB system only for imaging the femur, it was not readily adaptable to image a different tissue type. Instead, we performed an additional reproducibility study under the context of infection, where a higher motility and cell density are expected (S3 Fig). Two users independently tracked the neutrophils of the same IV-MLSM video from an infected animal following the semi-automated tracking protocol. The tracking remained reproducible under infected conditions, with high user agreement (ICC = 0.96 – 0.99). We modified the Results section to reflect this change:

“The major limitation of manual tracking is high inter-user variability. The two users assigned to analyze the same IV-MLSM video often identified different tracks. The lack of consistency is shown in the data distribution, where the median displacement and velocity of neutrophils differed by 25% and 22% respectively between the two users (S2 Fig). While these results were not statistically significant, such substantial numerical differences impede reliable conclusions. Furthermore, the inter-class correlation coefficient for manual tracking was as low as 0.85, indicating only a modest user agreement.

In contrast, our protocol yields reproducible tracking metrics from two users who independently analyzed three IV-MLSM videos. While there were minor differences in the tracks generated by both users (Fig 2, S3 Fig), we obtained robust inter-reader reliability (ICC > 0.96). The track metrics and data distribution are in good agreement (p > 0.05) (Fig 2C-2G, S3 Fig C-G).”

**S3 Fig. The tracking protocol resulted in low inter-person variability in generating tracks in the infected cases.** Two users independently analyzed IV-MLSM timelapse videos from an infected mouse and the generated track parameters were compared. Representative 2D IV-MLSM image with overlaid tracks generated by user 1 (A) and user 2 (B). Semiautomated quantification of neutrophil distance traveled (C), displacement (D), mean speed (E), mean velocity (F), and directionality (G), was performed and the data are presented with the median and interquartile range. (ns = not significant as determined by Mann-Whitney tests (n=67 tracks for user 1 and 65 tracks for user 2)).

***20. Future applications of the TWS machine learning tool should be outlined, providing a reasoned projection of its potential use in other directions.***

We have included this in the Discussion (page 16, lines 351-359), as well as in the response to Reviewer 1, comment 12.

**Do you want your identity to be public for this peer review?** For information about this choice, including consent withdrawal, please see our [Privacy Policy](https://urldefense.com/v3/__https:/www.plos.org/privacy-policy__;!!CGUSO5OYRnA7CQ!ZSsereCjBIF0VOM-YZtvonBCtzOhdgh_fhvidLkN9YGBdDa8G6JeOVRkufHN_xbSsDiHU9EMu-_GfpA1_E1ody0$).

Reviewer #1: **Yes:** yicheng zhao

Reviewer #2: No

**References:**

1.	Kienle K, Glaser KM, Eickhoff S, Mihlan M, Knöpper K, Reátegui E, et al. Neutrophils self-limit swarming to contain bacterial growth in vivo. Science (1979) [Internet]. 2021 Jun 18 [cited 2024 Feb 12];372(6548). Available from: https://www.science.org/doi/10.1126/science.abe7729

2.	Lázaro-Díez M, Chapartegui-González I, Redondo-Salvo S, Leigh C, Merino D, Segundo DS, et al. Human neutrophils phagocytose and kill Acinetobacter baumannii and A. pittii. Scientific Reports 2017 7:1 [Internet]. 2017 Jul 4 [cited 2024 Feb 12];7(1):1–11. Available from: https://www.nature.com/articles/s41598-017-04870-8

3.	Masters EA, Salminen AT, Begolo S, Luke EN, Barrett SC, Overby CT, et al. An in vitro platform for elucidating the molecular genetics of S. aureus invasion of the osteocyte lacuno-canalicular network during chronic osteomyelitis. Nanomedicine [Internet]. 2019 Oct 1 [cited 2024 Feb 13];21:102039. Available from: /pmc/articles/PMC6814543/

4.	Xie C, Ren Y, Weeks J, Rainbolt J, Kenney HM, Xue T, et al. Longitudinal intravital imaging of the bone marrow for analysis of the race for the surface in a murine osteomyelitis model. Journal of Orthopaedic Research® [Internet]. 2023 [cited 2023 Oct 29]; Available from: https://onlinelibrary.wiley.com/doi/full/10.1002/jor.25716

5.	Nishitani K, Sutipornpalangkul W, De Mesy Bentley KL, Varrone JJ, Bello-Irizarry SN, Ito H, et al. Quantifying the natural history of biofilm formation in vivo during the establishment of chronic implant-associated Staphylococcus aureus osteomyelitis in mice to identify critical pathogen and host factors. J Orthop Res [Internet]. 2015 Sep 1 [cited 2024 Feb 13];33(9):1311. Available from: /pmc/articles/PMC4529770/

6.	Hasenberg A, Hasenberg M, Männ L, Neumann F, Borkenstein L, Stecher M, et al. Catchup: a mouse model for imaging-based tracking and modulation of neutrophil granulocytes. Nature Methods 2015 12:5 [Internet]. 2015 Mar 16 [cited 2023 Aug 10];12(5):445–52. Available from: https://www.nature.com/articles/nmeth.3322

7.	Yipp BG, Petri B, Salina D, Jenne CN, Scott BNV, Zbytnuik LD, et al. Infection-induced NETosis is a dynamic process involving neutrophil multitasking in vivo. Nature Medicine 2012 18:9 [Internet]. 2012 Aug 26 [cited 2023 Dec 13];18(9):1386–93. Available from: https://www.nature.com/articles/nm.2847

8.	Legland D, Arganda-Carreras I, Andrey P. MorphoLibJ: integrated library and plugins for mathematical morphology with ImageJ. Bioinformatics [Internet]. 2016 Nov 15 [cited 2024 Mar 4];32(22):3532–4. Available from: https://dx.doi.org/10.1093/bioinformatics/btw413

9.	Ershov D, Phan MS, Pylvänäinen JW, Rigaud SU, Le Blanc L, Charles-Orszag A, et al. TrackMate 7: integrating state-of-the-art segmentation algorithms into tracking pipelines. Nature Methods 2022 19:7 [Internet]. 2022 Jun 2 [cited 2023 Aug 9];19(7):829–32. Available from: https://www.nature.com/articles/s41592-022-01507-1

10.	Salum P, Güven O, Aydemir LY, Erbay Z. Microscopy-Assisted Digital Image Analysis with Trainable Weka Segmentation (TWS) for Emulsion Droplet Size Determination. Coatings 2022, Vol 12, Page 364 [Internet]. 2022 Mar 9 [cited 2024 Feb 21];12(3):364. Available from: https://www.mdpi.com/2079-6412/12/3/364/htm

11.	Lormand C, Zellmer GF, Németh K, Kilgour G, Mead S, Palmer AS, et al. Weka Trainable Segmentation Plugin in ImageJ: A Semi-Automatic Tool Applied to Crystal Size Distributions of Microlites in Volcanic Rocks. Microscopy and Microanalysis [Internet]. 2018 Dec 1 [cited 2024 Feb 21];24(6):667–75. Available from: https://dx.doi.org/10.1017/S1431927618015428

12.	Stringer C, Wang T, Michaelos M, Pachitariu M. Cellpose: a generalist algorithm for cellular segmentation. Nature Methods 2020 18:1 [Internet]. 2020 Dec 14 [cited 2024 Feb 21];18(1):100–6. Available from: https://www.nature.com/articles/s41592-020-01018-x

13.	Berg S, Kutra D, Kroeger T, Straehle CN, Kausler BX, Haubold C, et al. ilastik: interactive machine learning for (bio)image analysis. Nature Methods 2019 16:12 [Internet]. 2019 Sep 30 [cited 2024 Feb 21];16(12):1226–32. Available from: https://www.nature.com/articles/s41592-019-0582-9
